# Outcomes of Fixed-Dose Radioactive Iodine Therapy in Hyperthyroidism and Optimization of Follow-Up After Treatment Failure With Low-Dose Antithyroid Medication

**DOI:** 10.7759/cureus.83812

**Published:** 2025-05-09

**Authors:** Panita Kantikool, Naphat Buraphanawibun

**Affiliations:** 1 Radiology/Nuclear Medicine, Ratchaburi Hospital, Muang Ratchaburi, THA; 2 Radiology, Galyani Vadhana Karun Hospital, Faculty of Medicine, Princess of Naradhiwas University, Muang, Narathiwas, THA

**Keywords:** duration follow up, euthyroid, failure treatment, fixed dose, hyperthyroid, radioactive iodine (rai) therapy, re-treatment

## Abstract

Introduction: Fixed-dose radioactive iodine (RAI) therapy is a common treatment for hyperthyroidism. However, the optimal follow-up duration and management strategies for patients experiencing treatment failure remain uncertain. This study evaluates the outcomes of fixed-dose RAI therapy in hyperthyroid patients and explores the appropriate follow-up duration for those requiring low-dose antithyroid drugs (ATDs) after initial treatment failure.

Methods: A retrospective cohort study was conducted, including 204 hyperthyroid patients who received their first fixed-dose RAI treatment at Ratchaburi Hospital, Na Muang, Ratchaburi, Thailand, between December 2022 and May 2024. Treatment outcomes were assessed six months post RAI. Among the 98 patients with treatment failure, those who remained euthyroid on low-dose ATDs were followed for up to nine months to evaluate long-term outcomes. The study analyzed remission rates, progression to hypothyroidism, and the need for additional RAI therapy using descriptive statistics and Kaplan-Meier survival analysis.

Results: Of the 204 patients, 106 (52%) achieved remission within six months post RAI. Among the 201 patients with Graves' disease, 104 (51.7%) achieved remission. Of the 98 patients with treatment failure, 40 remained euthyroid on low-dose ATDs. After nine months of extended follow-up, nine (37.5%) maintained euthyroid status, four (16.7%) developed hypothyroidism, and 11 (45.8%) required a second RAI treatment. The median time to treatment failure was 6.9 months, suggesting that some patients may stabilize without immediate re-treatment.

Conclusion: RAI therapy is effective for hyperthyroidism. Extended follow-up without rushing to a second RAI dose may be sufficient for patients who remain euthyroid on low-dose ATDs, helping reduce unnecessary radiation exposure. However, further prospective studies are needed to establish guidelines for optimal follow-up and re-treatment timing.

## Introduction

Hyperthyroidism is a condition characterized by excessive production of thyroid hormones, leading to systemic effects such as increased metabolic rate, unintentional weight loss, tachycardia or palpitations, excessive sweating, and fatigue [1]. The most common cause of hyperthyroidism is Graves’ disease, an autoimmune disorder that typically affects individuals aged 20-40 years and is more prevalent in females [2]. Another frequent etiology is toxic nodular goiter, a condition that progresses more gradually and is often observed in elderly patients.

If left untreated, hyperthyroidism can result in significant complications, particularly involving the cardiovascular system, and may lead to increased mortality [3]. Among the available treatment options, RAI therapy has long been established as a definitive and effective method for achieving remission. Fixed low-dose radioactive iodine (RAI) regimens are widely employed due to their simplicity, cost-effectiveness, and comparable outcomes to dosimetry-based approaches [4].

The 2016 American Thyroid Association (ATA) guidelines recommend assessing treatment outcomes approximately six months following RAI therapy to determine the need for further intervention, such as a second RAI dose or ongoing antithyroid drugs (ATD) therapy [1]. However, in clinical practice, a subset of patients maintains a euthyroid state on low-dose ATDs beyond the six-month mark without requiring immediate re-treatment. In such cases, clinicians may opt for extended monitoring, particularly when patients are hesitant to undergo repeat RAI therapy due to isolation protocols or concerns over radiation risks, including secondary malignancies and long-term hypothyroidism [1,5].

This study aims to evaluate the outcomes of fixed-dose RAI therapy in patients with hyperthyroidism in a hospital in Thailand where RAI treatment has only recently been introduced and medical resources, including specialized personnel and equipment, remain limited. It further explores the clinical course of patients with initial treatment failure who maintain a euthyroid state on low-dose ATDs, with the goal of identifying appropriate follow-up strategies in settings where immediate re-treatment may not be feasible. As existing literature on this specific group is limited, the findings from this study may offer valuable insights to support clinical decision-making in similar resource-constrained environments.

## Materials and methods

This was a retrospective cohort study conducted in Ratchaburi Hospital, Muang, Ratchaburi, Thailand. The study was approved by the Human Research Ethics Committee of Ratchaburi Hospital (approval number: COA-RBHEC 003/2025).

Inclusion and exclusion criteria

The study included adults (≥18 years) diagnosed with hyperthyroidism who received their first fixed-dose RAI treatment (10 mCi) at Ratchaburi Hospital between December 2022 and May 2024. Eligible patients had discontinued ATDs three to seven days before RAI therapy and resumed them three to seven days afterward. Follow-up visits were conducted every two to three months, and treatment outcomes were assessed at six months. Exclusion criteria included a history of thyroid surgery, insufficient follow-up (<6 months), or loss to follow-up in the failure group before nine months.

Definitions

Remission was defined as achieving a euthyroid or hypothyroid state based on thyroid hormone levels without the use of ATDs. Treatment failure was defined as persistent biochemical hyperthyroidism or continued need for ATDs beyond six months. 

Data collection

Patients who maintained normal thyroid function on low-dose methimazole (MMI) (≤5 mg/day) or propylthiouracil (PTU) (≤50 mg/day) were monitored for up to nine months. Clinical records were reviewed to obtain data on patient demographics, thyroid function tests, treatment history, and follow-up after fixed-dose RAI therapy.

Data analysis

All patient data were fully anonymized prior to analysis. Continuous variables were reported as means with standard deviations (SD) or medians with minimum and maximum values. Categorical variables were presented as frequencies and percentages. Kaplan-Meier analysis was used to evaluate follow-up duration and time to treatment failure.

## Results

Baseline characteristics of patients

A total of 204 patients with hyperthyroidism who received their first fixed-dose RAI therapy at Ratchaburi Hospital between December 2022 and May 2024 were included in the analysis. The majority of patients were female (n=155; 76.0%), and most were diagnosed with Graves’ disease (n=201; 98.5%). The mean age was 41.0 ± 9.5 years. Prior to RAI, 182 (89.2%) patients were treated with MMI, while a smaller proportion received PTU. Most patients (n=114; 61.3%) had elevated thyroid hormone levels at the time of treatment. The baseline characteristics of patients are summarized in Table 1.

**Table 1:** Baseline characteristics of patients receiving first fixed-dose RAI therapy (N=204) Data presented as frequency (percentage), except age, which is presented as mean±SD TMNG: toxic multinodular goiter; RAI: radioactive iodine; NA: not available

Variables	Frequency (Percentage)
Sex	
Male	49 (24.0%)
Female	155 (76.0%)
Age (years), mean±SD	41.0±9.5
Type of disease	
Graves’ disease	201 (98.5%)
TMNG	3 (1.5%)
Medication	
Methimazole	182 (89.2%)
Propylthiouracil	16 (7.8%)
NA	6 (2.9%)
Thyroid hormone level before treatment	
Normal and low	72 (38.7%)
High	114 (61.3%)
NA	18 (8.8%)

Treatment response and follow-up period

At six months after the initial fixed-dose RAI therapy, 106 patients (52%) achieved remission, defined as either euthyroid or hypothyroid status without the need for ATDs, based on clinical assessment and thyroid function tests. When considering only patients with Graves’ disease, 104 out of 201 patients (51.7%) achieved remission. The remaining 98 (48%) patients were categorized as treatment failures. Among these, 40 patients maintained a euthyroid state while on low-dose ATDs and were selected for extended follow-up. Of this subgroup, 24 (60%) patients completed the full nine-month follow-up, while 16 (40%) patients were excluded due to loss to follow-up. The baseline characteristics of the 24 patients who completed extended monitoring are summarized in Table 2.

**Table 2:** Characteristics of patients with initial treatment failure who completed follow-up (N=24) Data presented as frequency (percentage), except age, which is presented as mean±SD

Variables	Frequency (Percentage)
Sex	
Male	6 (25.0%)
Female	18 (75.0%)
Age (years), mean±SD	40±24.7
Type of disease	
Graves’ disease	24 (100%)
Medication	
Methimazole	18 (75.0%)
Propylthiouracil	6 (25.0%)

**Figure 1:**
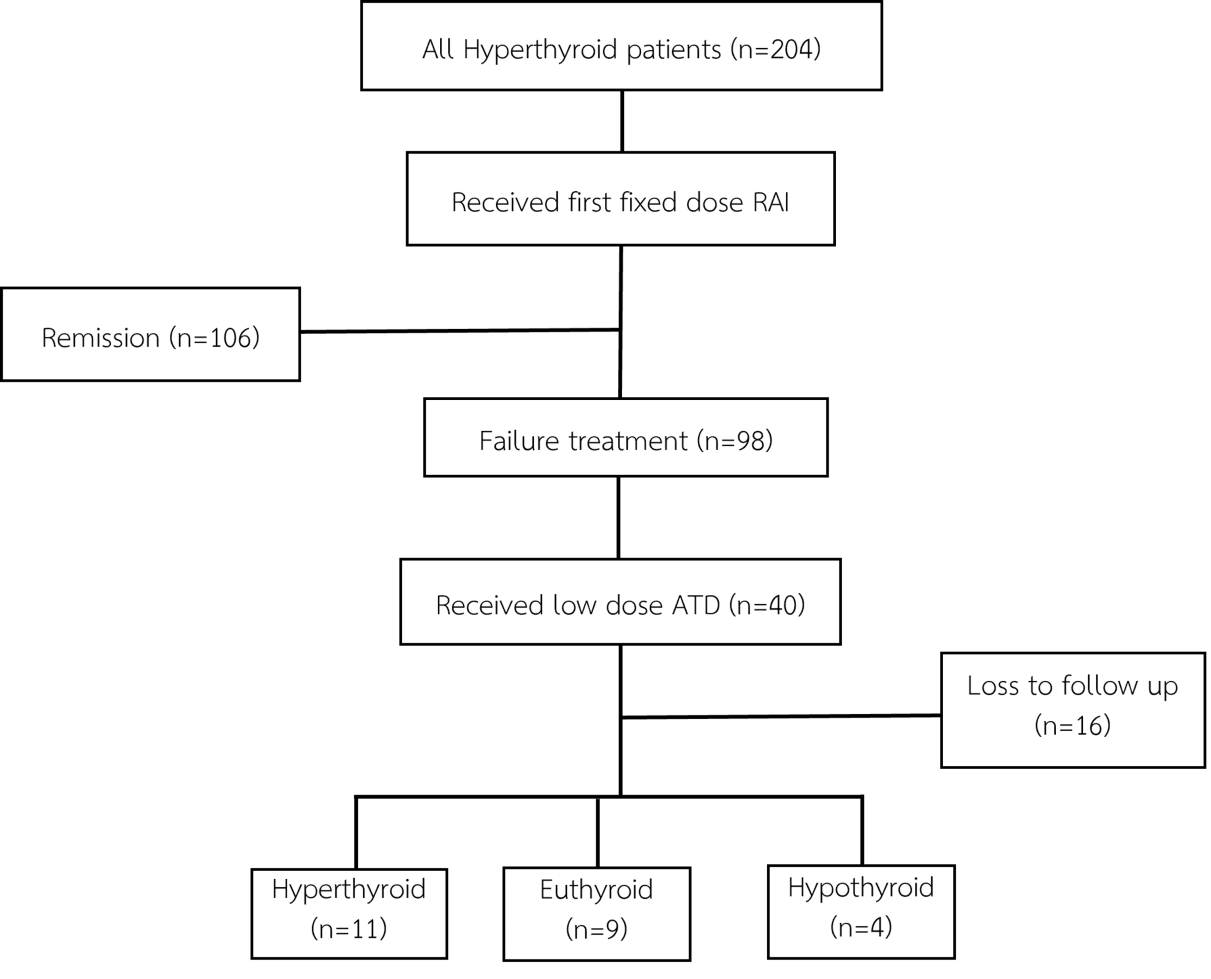
Flowchart depicting the distribution of patients RAI: radioactive iodine; ATD: antithyroid drug

The majority of these 24 patients were female (n=18; 75%), with a mean age of 40.0 ± 24.7 years. All patients were diagnosed with Graves' disease. Most were treated with MMI (n=18; 75%), while the remaining six (25%) received PTU. Outcomes among these patients were as follows: nine (37.5%) remained euthyroid without requiring further intervention, four (16.7%) developed overt hypothyroidism and initiated levothyroxine therapy, and 11 (45.8%) experienced recurrent hyperthyroidism, necessitating a second course of RAI therapy (Figure 1).

**Figure 2:**
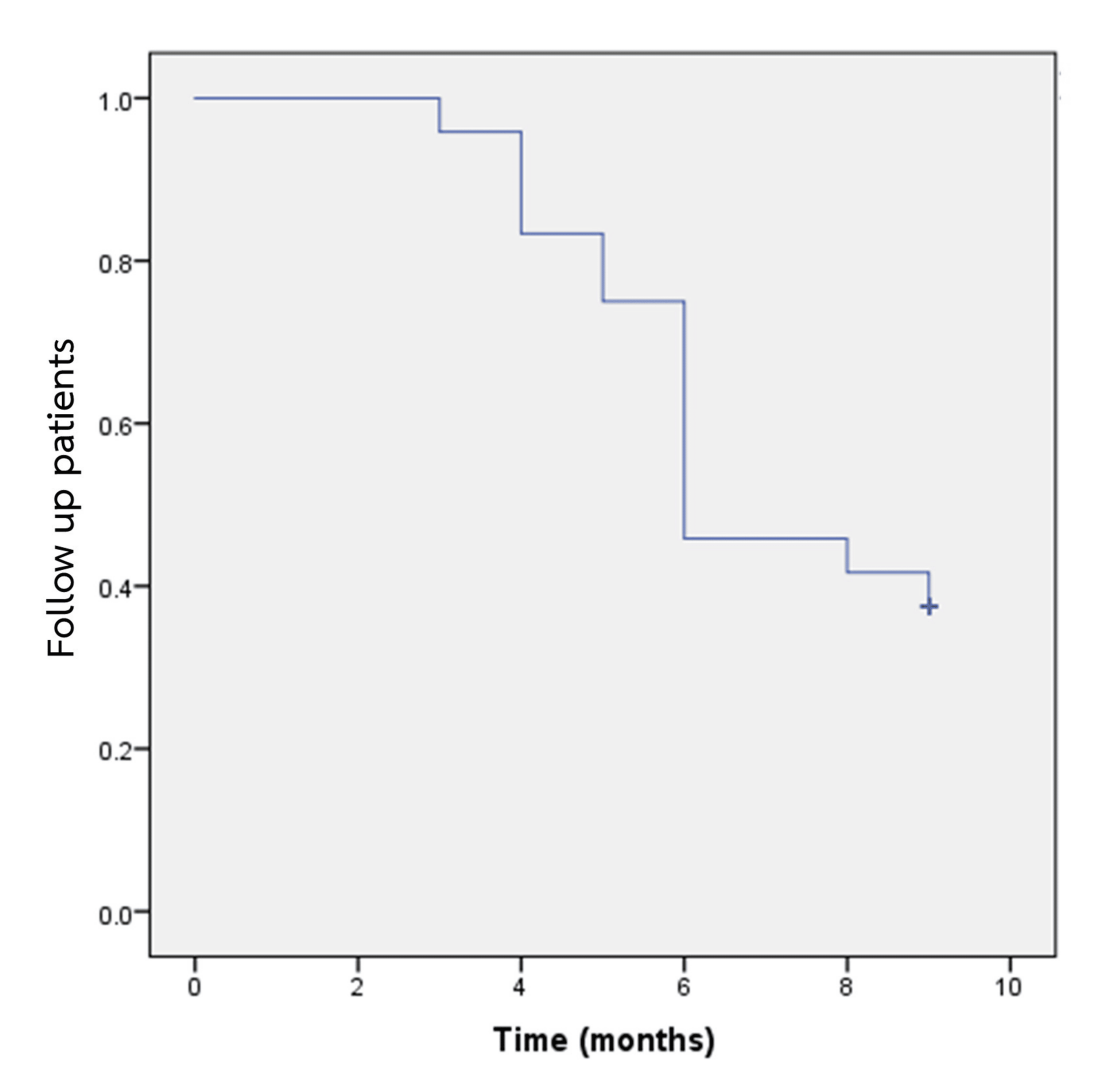
Kaplan-Meier curve for follow-up patients with low-dose antithyroid drugs

Kaplan-Meier curve analysis demonstrated that the median time from the initial RAI therapy to treatment failure requiring a second RAI was 6.9 months. Including the initial post-treatment period, the total median time to re-treatment was 12.9 months. These findings are illustrated in Figure 2.

## Discussion

RAI therapy is widely recognized as the preferred treatment for hyperthyroidism, especially in patients with Graves' disease, due to its ability to effectively induce remission and convenience compared to ATD therapy or surgery [6,7].

In this study, the overall remission rate following fixed-dose RAI therapy was 104 (52%), which is relatively lower compared to previously published studies. Khan et al. (2023) reported a remission rate of 83.1% (69/83 patients) among hyperthyroid patients treated with fixed-dose RAI, attributing the higher success rate to early intervention and careful patient selection [8]. Similarly, Shinto et al. (2010) observed a remission rate of approximately 98.8% (156/158 patients) in a South Indian cohort, with treatment outcomes significantly influenced by gland size and disease severity [9]. El-Kareem et al. (2014), in a 10-year retrospective study from Egypt, reported an overall remission rate of 59.8% (192/321) following first fixed doses of RAI, particularly when higher doses (12 mCi) were used compared to lower doses (8 mCi), suggesting a dose-dependent treatment response [10]. Nair et al. (2022) also reported a high remission rate of 85% (57/67) in a single-center study, with the majority of patients responding well to a single fixed dose of RAI [11]. 

The comparatively lower remission rate observed in the present study may be associated with differences in baseline patient characteristics. A substantial proportion of patients in the current cohort presented with larger thyroid gland volumes and elevated baseline thyroid hormone levels, factors that have been previously identified as negatively impacting the efficacy of RAI therapy. These findings align with results from studies conducted in Thailand. Kiatkittikul et al. (2021) demonstrated that adjusting RAI doses based on thyroid gland size resulted in improved treatment outcomes, with a success rate of 50.0% (89/178 patients) (95%CI: 42.4-57.6%), emphasizing the significance of anatomical factors in optimizing therapeutic efficacy [12]. Similarly, Rattanamanee and Samphantharat (2022) reported a success rate of 49.4% (743/1502 patients) within six months, which increased to 60.3% (859 patients) within 12 months, highlighting the importance of individualized treatment approaches [13]. Regional variations in dietary iodine intake, individual iodine uptake, and differences in treatment protocols may influence the remission rates observed across studies.

According to the 2016 American Thyroid Association (ATA) guidelines, patients who receive RAI therapy should be monitored for a period of six months before considering retreatment. However, due to institutional limitations at our center, where frequent administration of RAI is not feasible and there are patient-related factors such as financial constraints or challenges related to hospital access, immediate retreatment was not always possible. As a result, a subset of 40 patients who remained on low-dose ATDs and achieved a euthyroid state at six months following RAI treatment were observed for an additional nine months. During this extended follow-up period, nine (37.5%) maintained euthyroid status, four (16.7%) developed overt hypothyroidism requiring thyroid hormone replacement, and 11 (45.8%) experienced recurrent or worsening hyperthyroidism necessitating a second dose of RAI. Kaplan-Meier analysis demonstrated that the median time from treatment failure after initial RAI therapy was 6.9 months, corresponding to approximately 12.9 months after the first RAI treatment. This aligns with prior studies indicating that a subset of patients can maintain euthyroid status for extended periods despite initial RAI failure.

These results highlight the variable and often delayed nature of the response to RAI therapy. Thyroid dysfunction may take several months or even years to manifest. For example, Roque et al. (2020) reported cure rates increasing from 44% at six months to 94% at 10 years, with a mean time to hypothyroidism of 2.7 ± 2.4 years [14]. Similarly, Metso et al. (2004) found that some patients remained euthyroid for up to two years without additional RAI therapy [15]. In addition, Kim et al. (2022) reported that 81 (42%) patients with persistent hyperthyroidism post-RAI eventually achieved remission [16].

These findings emphasize the individual variability in thyroid response to RAI treatment; some patients may retain sufficient thyroid function for an extended period, delaying the onset of hypothyroidism [15-17]. This suggests that delaying re-treatment beyond six months may allow for spontaneous remission in some patients, potentially reducing the need for further RAI doses and minimizing unnecessary radiation exposure.

Limitations of the study

Certain limitations should be considered. Approximately 40% of the initial cohort was lost to follow-up, mainly because our hospital is a tertiary care referral center, and patients often return to their primary hospitals after receiving specific treatments. This loss to follow-up may have introduced selection bias and resulted in an overestimation of treatment failure rates. Given the retrospective design, variability in follow-up intervals, and incomplete data, the outcome assessment may have been influenced. Further prospective studies are needed to confirm these findings and refine follow-up protocols, especially concerning the optimal timing for re-treatment.

## Conclusions

This study evaluated the outcomes of fixed-dose RAI therapy for hyperthyroidism and examined the optimal follow-up duration for patients who experienced treatment failure while on low-dose ATDs. Our findings indicate that while a significant proportion of patients achieved remission after initial RAI therapy, some did not respond successfully. Among those with treatment failure, a subset remained euthyroid with low-dose ATDs beyond the standard six-month evaluation period. Extending follow-up to nine months revealed that some patients stabilized without additional interventions, some eventually achieved remission, while others ultimately required a second RAI therapy.

These results highlight the individual variability in thyroid response to RAI treatment and suggest that delaying re-treatment beyond six months in certain patients may offer the opportunity for spontaneous remission, potentially reducing unnecessary radiation exposure. However, the limitations of this retrospective study, including loss to follow-up and incomplete data, warrant caution in interpreting the results. Further prospective studies with consistent follow-up intervals are needed to refine follow-up protocols and establish evidence-based guidelines for optimizing treatment timelines and improving long-term management strategies for hyperthyroid patients undergoing RAI therapy.
